# Red blood cell oxidative stress impairs oxygen delivery and induces red blood cell aging

**DOI:** 10.3389/fphys.2014.00084

**Published:** 2014-02-28

**Authors:** Joy G. Mohanty, Enika Nagababu, Joseph M. Rifkind

**Affiliations:** Molecular Dynamics Section, Laboratory of Molecular Gerontology, National Institute on AgingBaltimore, MD, USA

**Keywords:** red blood cells, oxidative Stress, deformability, heme degradation, cellular aging

## Abstract

Red Blood Cells (RBCs) need to deform and squeeze through narrow capillaries. Decreased deformability of RBCs is, therefore, one of the factors that can contribute to the elimination of aged or damaged RBCs from the circulation. This process can also cause impaired oxygen delivery, which contributes to the pathology of a number of diseases. Studies from our laboratory have shown that oxidative stress plays a significant role in damaging the RBC membrane and impairing its deformability. RBCs are continuously exposed to both endogenous and exogenous sources of reactive oxygen species (ROS) like superoxide and hydrogen peroxide (H_2_O_2_). The bulk of the ROS are neutralized by the RBC antioxidant system consisting of both non-enzymatic and enzymatic antioxidants including catalase, glutathione peroxidase and peroxiredoxin-2. However, the autoxidation of hemoglobin (Hb) bound to the membrane is relatively inaccessible to the predominantly cytosolic RBC antioxidant system. This inaccessibility becomes more pronounced under hypoxic conditions when Hb is partially oxygenated, resulting in an increased rate of autoxidation and increased affinity for the RBC membrane. We have shown that a fraction of peroxyredoxin-2 present on the RBC membrane may play a major role in neutralizing these ROS. H_2_O_2_ that is not neutralized by the RBC antioxidant system can react with the heme producing fluorescent heme degradation products (HDPs). We have used the level of these HDP as a measure of RBC oxidative Stress. Increased levels of HDP are detected during cellular aging and various diseases. The negative correlation (*p* < 0.0001) between the level of HDP and RBC deformability establishes a contribution of RBC oxidative stress to impaired deformability and cellular stiffness. While decreased deformability contributes to the removal of RBCs from the circulation, oxidative stress also contributes to the uptake of RBCs by macrophages, which plays a major role in the removal of RBCs from circulation. The contribution of oxidative stress to the removal of RBCs by macrophages involves caspase-3 activation, which requires oxidative stress. RBC oxidative stress, therefore, plays a significant role in inducing RBC aging.

## Introduction

### The red blood cell oxidative stress

The functional role of Red Blood Cells (RBCs) is the transport of oxygen from the lungs to the tissues providing all cells with the required oxygen. In the circulation, RBCs are continuously exposed to both endogenous and exogenous sources of reactive oxygen species (ROS) that can damage the RBC and impair its function. To minimize the effect of these ROS and the resultant oxidative stress, RBCs have an extensive antioxidant system involving both non-enzymatic low molecular weight antioxidants like glutathione and ascorbic acid and enzymatic antioxidants including superoxide dismutase, catalase (Gonzales et al., 1984), glutathione peroxidase (Nagababu et al., 2003) and peroxiredoxin-2 (PRDX-2) (Lee et al., 2003; Nagababu et al., 2013).

ROS are highly reactive and many of the ROS released from neutrophils and macrophages into the plasma are neutralized before they can be taken up by RBCs. However, particularly in the microcirculation, where the RBCs are in close proximity and even make contact with the vasculature (Rifkind et al., 1997; Nagababu and Rifkind, 1998), ROS released from neutrophils (Aoshiba et al., 1999), macrophages and endothelial cells are taken up by RBCs. Upon entry into RBC cytoplasm, they are for the most part neutralized by the cytosolic antioxidant system. In fact, hydrogen peroxide added to RBCs rapidly reacts with catalase being converted to oxygen without any oxidation of hemoglobin (Hb). In addition, endogenous ROS are continuously generated by the slow autoxidation of Hb (Abugo and Rifkind, 1994), which produces methemoglobin (that can no longer carry oxygen) and superoxide that rapidly dismutates to form hydrogen peroxide. The bulk of these ROS are also neutralized by the RBC cytosolic antioxidants. However, the ability of the antioxidant system to neutralize the endogenous ROS is limited as the blood flows through the microcirculation and Hb becomes partially oxygenated. Partial oxygenation results in a Hb conformational change with certain unique properties (see below). Thus, there is a dramatic increase in the rate of Hb autoxidation for partially oxygenated Hb (Abugo and Rifkind, 1994; Balagopalakrishna et al., 1996). At the same time an increase in the affinity of partially oxygenated Hb for the RBC membrane (that is appreciably greater than for fully oxygenated or fully deoxygenated Hb (Cao et al., 2009), limits the efficiency of the antioxidant system (that is primarily cytosolic) from neutralizing the ROS formed at the membrane. This pool of un-neutralized ROS in the RBC has been shown to (1) damage the RBC membrane (Nagababu et al., 2008; Barodka et al., 2013b) impairing the flow of RBCs through the microcirculation and the delivery of oxygen to the tissues and to (2) be transferred to cells, which come in contact with RBCs resulting in tissue damage that induces inflammation (Kiefmann et al., 2008; Huertas et al., 2013).

Recent studies indicate that RBCs also contain NADH oxidases, which can generate endogenous ROS (George et al., 2013). The initial demonstration of RBC NADH oxidase and its potential physiological ramification involved sickle cell disease. However, some forms of NADH oxidase were also detected in normal RBCs. Additional studies are necessary to determine the functional effect of NADH oxidase in normal cells and any possible effect on cellular aging. As found for Hb generated ROS, the ROS generated in the cytoplasm would under most conditions be neutralized by the RBC antioxidant system. However, George et al has found that one of the NADH oxidase isoforms present in RBCs is located on the membrane. The potential effect of this isoform of NADH oxidase needs to be further investigated during cellular aging in normal RBCs.

### The level of heme degradation, a measure of RBC oxidative stress

The presence of antioxidant enzymes as well as the relative instability of ROS makes it very difficult to quantitate the pool of un-neutralized ROS that reflect RBC oxidative stress. This affects both the RBC and other cells the RBC comes in contact with. As a solution for this problem we have found that a small fraction of the non-neutralized hydrogen peroxide degrades the protoporphyrin producing fluorescent heme degradation products (HDPs) that can be detected even at very low concentrations (Nagababu and Rifkind, 1998). These HDPs are also not neutralized by the RBC antioxidant systems and are, therefore, much more stable.

These HDPs were originally detected (Nagababu and Rifkind, 1998) when a 10 fold excess of hydrogen peroxide was added to oxyhemoglobin (oxyHb). At this concentration, in addition to the formation of methemoglobin (metHb), ~5% of the hemes were degraded producing two fluorescent products. One of those has an excitation wavelength of 321 nm and emission wavelength in the region of 465 nm and the second product has an excitation wavelength of 460 nm and emission wavelength in the region of 525 nm. Confirmation that these fluorescent bands are attributed to HDPs is based on the observation that the same fluorescent bands were obtained when hydrogen peroxide reacted with heme or hemin, although these reactions required much higher levels of hydrogen peroxide. In addition, the excitation and emission wavelengths for these bands were distinct from those of globin fluorescent amino acids like tryptophan, tyrosine or di-tyrosine (Teale, 1960; Giulivi and Davies, 1993) as well as free protoporphyrin IX. Thus, these fluorescent bands originating from heme degradation are considered as markers of RBC oxidative stress.

The mechanism for the formation of these degradation products was shown to require (Nagababu and Rifkind, 2000) an initial reaction with hydrogen peroxide producing Fe(IV) ferrylhemoglobin (ferrylHb). The formation of ferrylHb was confirmed by showing that sodium sulfide, which reacts with ferrylHb, inhibits the formation of the HDPs. FerrylHb then reacted with a second molecule of hydrogen peroxide. The requirement for this hydrogen peroxide was demonstrated by the finding that catalase added after the ferrylHb had formed, inhibited the formation of HDPs. This second molecule of hydrogen peroxide produced metHb and a superoxide radical, which was retained in the heme pocket and was detected by electron paramagnetic resonance. The retention of this superoxide in the heme pocket much longer than the superoxide formed during Hb autoxidation (see above) facilitates a reaction of the superoxide with the porphyrin initiating the heme degradation process.

The significance of this reaction is indicated by the demonstration that the same HDPs are generated from the low levels of hydrogen peroxide constantly being produced by the dismutation of superoxide released (Nagababu and Rifkind, 2000) during the autoxidation of purified Hb.

In studies with intact RBCs, we found that we can detect the same fluorescent band (Ex: 321 nm) as in HDPs described above in any fresh RBC sample (Nagababu et al., 2010). We further demonstrated that the amount of heme degradation increased for RBCs in circulation for a longer period of time (older RBCs) (Nagababu and Rifkind, 2004). These results indicate that HDPs are produced in the RBCs even though they have an extensive antioxidant system that should react with any amount of hydrogen peroxide formed.

This paradox is explained by the finding that in RBCs, almost all of the HDPs are located on the membrane (Nagababu et al., 2010). To rule out the uptake of cytoplasmic HDPs by the more hydrophobic membrane, we incubated Hb reacted with hydrogen peroxide with RBC membranes and found no increase in the level of membrane fluorescent products over a period of 12 h. These results thus indicate that the HDPs generated in the RBC are formed on the RBC membrane and not in the cytoplasm.

Hb is known to bind to the cytoplasmic end of band 3 (Evans and Fung, 1972; Shaklai et al., 1977a,b) present in the membrane. It has been documented that deoxyhemoglobin (deoxyHb) has an appreciably higher affinity for band 3 than oxyHb. This difference has been attributed to changes in the subunit interactions, which facilitate interactions between the cytoplasmic end of band 3 and Hb. While these earlier studies have compared fully oxygenated and fully deoxygenated Hb with known differences in quaternary structure, we have been involved in the studies with partially oxygenated Hb present in RBCs in the microcirculation. Evidence for a distinct conformation for partially oxygenated Hb was initially demonstrated by the dramatic increase in the rates of autoxidation when Hb is partially oxygenated (Abugo and Rifkind, 1994; Balagopalakrishna et al., 1996). Recent studies (Cao et al., 2009) imply that this same conformational change, which alters the interactions between Hb subunits, also has a dramatic effect on their affinity for the RBC membrane. We, thus, found that low levels of nitrite/NO reacted Hb present in fully deoxygenated RBCs have an affinity >100-fold greater for the RBC membrane than deoxyHb. We have attributed this to the nitrite/NO bound fraction of the Hb that is partially liganded, with properties similar to that of partially oxygenated Hb.

Thus, the partially oxygenated Hb is responsible for the bulk of the ROS formed by Hb autoxidation. However, with the elevated affinity of this fraction of Hb for the RBC membrane, the superoxide and hydrogen peroxide formed during the autoxidation of this Hb, is relatively inaccessible to the cytosolic catalase and superoxide dismutase. So, an appreciable fraction of these ROS can react with the Hb before being neutralized by the RBC antioxidant system. Unlike the potential for such reactions with endogenously generated ROS, the addition of exogenous hydrogen peroxide is immediately transported into the RBC before it can react with membrane bound Hb and is neutralized predominantly by cytosolic catalase (Nagababu et al., 2010). While catalase does not seem to be able to compete with Hb in reacting with the pool of hydrogen peroxide generated on the membrane, glutathione peroxidase (Nagababu et al., 2003) and PRDX-2 (Nagababu et al., 2013) may play a role in neutralizing ROS generated on the RBC membrane. Glutathione peroxidase is known to react with membrane ROS (Horton and Fairhurst, 1987) and its inhibition was found to dramatically increase the formation of HDPs. Although PRDX-2 is primarily a cytosolic enzyme, ~5% of it is membrane associated (Moore et al., 1991; Low et al., 2004). The neutralization of membrane generated ROS by PRDX-2 was postulated to explain an increase in heme degradation in PRDX-2 knockout mice (Nagababu et al., 2013).

### RBC oxidative stress impairs cellular deformability necessary for effective oxygen transport and delivery

RBCs are larger than the capillary diameter in the microcirculation (Pries et al., 1996). Blood flow, therefore, requires that the discoid RBCs deform to squeeze through these capillaries and deliver oxygen to the tissues. The ROS generated on the RBC membrane through Hb autoxidation are ideally located to react with membrane lipids and proteins producing lipid peroxidation and modified membrane proteins that can affect the membrane structure.

Exogenous xanthine oxidase, which generates superoxide, has been shown to affect the lipids by increasing their peroxidation. It has, however, been shown that this lipid oxidative damage does not affect the RBC deformability (Gurbuz et al., 2004). Nevertheless, it has been shown that deformability of RBCs is impaired as a result of treatment of RBCs by a number of reagents associated with oxidative stress including hydrogen peroxide, t-butyl hydroperopxide, cumene hydroperoxide, verapamil and ascorbate (Kuypers et al., 1990; Kim et al., 2008). Damage to membrane proteins is, thus, presumably responsible for the impaired cellular deformability associated with oxidative stress. The role of protein damage in producing impaired deformability is consistent with a dominant role for the membrane cytoskeleton (Suzuki et al., 2007) in regulating RBC deformability. There are a number of specific cases where it has been shown that damage to membrane and cytoskeletal proteins affects deformability (Chasis and Mohandas, 1986; Cluitmans et al., 2012; Grau et al., 2013).

A linear relationship between deformability and RBC oxidative stress, as measured by the level of HDPs (see above) has been found in studies involving sickle cell disease where subjects under crisis and not under crisis as well as those with sickle cell trait were compared (Barodka et al., 2013b). Comparing all of these subjects, a highly significant correlation [*N* = 34; *R*= (−)0.68; *p* < 0.0001] was found between RBC deformability and the level of HDPs. We also found that with transgenic mice lacking PRDX-2 and superoxidase-2 (SOD2) (Mohanty et al., 2013; Nagababu et al., 2013), there were appreciable increases in heme degradation that coincided with a decrease in RBC deformability.

Despite this correlation, by comparing the average changes in deformability and heme degradation for normal healthy subjects, subjects with sickle cell trait, subjects with sickle cell disease not undergoing crisis and those undergoing crisis, we were able to delineate the different factors that contribute to the changes in heme degradation and RBC deformability. Heme degradation, which is primarily affected by the instability of the Hb, increased even for subjects with sickle cell trait, because of the increase in unstable sickle cell Hb. On the other hand, the RBC deformability in addition to being affected by oxidative stress was also affected by the changes in Hb aggregation that occurs during sickle cell crisis (see below).

The contribution of oxidative stress that does not necessarily involve Hb autoxidation to deformability is indicated by caspase-3. Caspase 3, is activated in the RBC by oxidative reactions, such as the reaction with tertiary butyl hydroperoxide, has been shown to partially degrade band 3 (Mandal et al., 2003; Clementi et al., 2007). This reaction has been shown to induce the exposure of phosphatidylserine (PS, usually located on the inner leaflet of the RBC membrane) to the outer surface (Mandal et al., 2005). This dramatic rearrangement of the membrane has been shown to involve a concomitant decrease in deformability (Fens et al., 2012).

Oxidative stress has also been shown to inhibit Ca-ATPase (Samaja et al., 1990; Kiefer and Snyder, 2000), which is responsible for limiting the intracellular concentration of calcium. Enhanced intracellular calcium has several effects that can affect deformability. This includes activation of the Gardos channel resulting in leakage of potassium from the RBC affecting cation homeostasis (Ney et al., 1990; Barodka et al., 2013a) causing shrinkage of the cell and impaired deformability. Calcium also activates calpain that can degrade additional proteins on the membrane (Redding et al., 1991).

Despite the demonstrated relationship between RBC deformability and oxidative stress, deformability can be affected by other processes that are not associated with oxidative stress. Although calcium induced shrinkage is associated with oxidative stress, membrane microvesiculation is a regulated process that is accelerated in older cells (Willekens et al., 2008) and is not thought to involve oxidative stress. It is, however, responsible for the increase in cell density coupled with a decrease in the cellular deformability and flexibility (Bartosz, 1991; Abugo and Rifkind, 1994; Wang et al., 2010). In addition to regulated vesiculation, any change that affects the volume of the cell and the excess surface area, will affect the deformability of the RBCs.

There are, in addition, changes in the intracellular content that can affect deformability independent of oxidative stress. A documented case of such a deformability change involves sickle cell disease (Barodka et al., 2013b). We have alluded to the correlation between heme degradation and impaired deformability for sickle cell disease which is attributed to the instability of sickle cell Hb (see above). However, a careful analysis of the data indicates that deformability is also affected by the aggregation of Hb that involves the structural change in deoxygenated Hb for sickle cell Hb, which triggers sickling of the RBC during sickle cell crisis. In this case the sickling process directly impairs the ability of the cell to deform.

### Contribution of RBC oxidative stress to RBC aging

The RBC, continuously undergoing normoxic and hypoxic cycling, is constantly exposed to oxidative insults during its 120 day life-span that results in continuous biochemical, physical, and structural changes. These changes impair the ability of the RBC to transport oxygen and eventually trigger its removal from the circulation by the reticulo-endothelial system. The reticulo-endothelial system involves the mononuclear phagocytic cells primarily in the spleen, but also in the liver and lymph nodes.

The processes responsible for the actual triggering of the removal have been extensively studied (Ajmani and Rifkind, 1998; Barvitenko et al., 2005; Rogers et al., 2009; Antonelou et al., 2010). Many of the processes involve oxidative stress.

The RBC membrane band 3 is the dominant integral trans-membrane protein. It has several crucial functions including: (1) the maintenance of anion homeostasis, (2) providing a link between the membrane and the cytoskeleton responsible for maintaining the cell shape and (3) providing for the interaction of a number of cytosolic proteins with the membrane via the amino terminal region that protrudes into the cytosol. This region of band 3 binds competitively both Hb, and a number of glycolytic enzymes (Mohandas and Gallagher, 2008). The changes in Hb binding to band 3 as a function of the Hb oxygenation, therefore, couple Hb oxygenation, Hb autoxidation, glycolysis and ATP production (De Rosa et al., 2008).

Oxidative damage to band 3 has been linked to RBC aging including the exposure of senescent specific neo-antigens that bind autologous IgG triggering RBC removal (Kay, 1993). IgG binding has also been linked to band 3 clusters, which is triggered by the binding of denatured oxidized Hb (hemichromes) to band 3 (Low et al., 1985; Rettig et al., 1999; Ferru et al., 2011).

Caspase-3 activation, which involves oxidative stress (see above), also cleaves the cytoplasmic end of band 3 (Mandal et al., 2003) affecting the interactions of band 3 with cytosolic proteins as well as the linkage to ankyrin and the cytoskeleton, which also induces PS exposure (Grey et al., 2012) (see below).

Membrane micro-vesiculation is a process that accelerates in the formation of older cells (Willekens et al., 2008) (see above). These changes limit the ability of the RBC to maintain the highly deformable biconcave shape necessary to pass through narrow pores, thus contributing to their removal from circulation. While cell shrinkage and vesiculation can be induced by various factors, some of which may not involve oxidative stress, the shrinkage associated with potassium leakage through the Gardos channel is triggered by oxidative stress. This process is initiated by damage to Ca-ATPase, which maintains a low intracellular concentration of free calcium ions (Larsen et al., 1981). Damage to Ca-ATPase is responsible for the age induced increase in intracellular calcium and is generated by oxidative damage to the ATPase (Samaja et al., 1990; Kiefer and Snyder, 2000). The increase in intra-cellular calcium activates the Gardos channel causing the leakage of potassium from the cell resulting in cell shrinkage and impaired deformability (Brugnara, 1993; Foller et al., 2008b).

An increase in intracellular calcium also activates calpain, transglutaminase-2 and some caspases that can degrade/crosslink cytoskeleton proteins (Redding et al., 1991). It also inhibits phosphotyrosine phosphatase increasing band 3 phosphorylation (Zipser et al., 2002).

The RBC lipid bilayer contains an asymmetric distribution of phospholipids with PS being maintained on the inner surface of the membrane by the competition between Scramblase, which randomizes the distribution and Flippase, which internalizes the PS. Coupled with an increase in Sphingomyelinase that increases ceramide, increased intracellular calcium has been linked to the exposure of PS and to a reduction in Flippase activity (Burger et al., 2013), that triggers the interaction of RBCs with macrophages and eryptosis (Daleke, 2008; Foller et al., 2008a; Weiss et al., 2011). Despite the important role of macrophages in the removal of RBCs,it is not clear that the interaction of RBCs with macrophages is responsible for the removal of aged RBCs from circulation (Dasgupta et al., 2008; Saxena et al., 2012).

## Conclusion

Partially oxygenated Hb molecules formed in the RBCs in microcirculation, when oxygen is being transported by them to the tissues, have an elevated affinity for the RBC membrane and have an increase in autoxidation producing ROS that are not completely neutralized by the RBC antioxidant system. This source of RBC oxidative stress is involved in a number of the factors that contribute to RBC aging and the removal of RBCs from the circulation. This oxidative process, thus, explains the dominant role of oxidative stress in RBC aging.

### Conflict of interest statement

The authors declare that the research was conducted in the absence of any commercial or financial relationships that could be construed as a potential conflict of interest.
